# Screening and Phasewise Management of Burn Injuries

**DOI:** 10.7759/cureus.54915

**Published:** 2024-02-26

**Authors:** Neha N Sarda, Snehlata Hingway

**Affiliations:** 1 Department of Medicine and Surgery, Jawaharlal Nehru Medical College, Datta Meghe Institute of Higher Education and Research, Wardha, IND; 2 Department of Pathology, Jawaharlal Nehru Medical College, Datta Meghe Institute of Higher Education and Research, Wardha, IND

**Keywords:** inflammation, keloid and hypertrophic scar, fluid resuscitation, hypermetabolism, burn injuries

## Abstract

Thermal, electrical, chemical, or electromagnetic radiation can cause painful wounds or burns. Spilling hot liquids onto the skin can also cause these kinds of injuries. The two biggest factors contributing to burn injuries in the elderly are smoking and exposure to open flames, while scalding is the primary cause of burn damage in children. Newborns and the elderly make up the majority of burn casualties. In India, there are estimated to be 6-7 million burn cases per year. The high incidence is attributed to the population's illiteracy, poverty, and lack of awareness of safety. The problem is made much worse by the fact that basic and secondary healthcare levels do not provide systematic burn care. Coagulation necrosis is caused by denaturing proteins due to heat from burns. Platelets clump together, arteries narrow, and partly perfused tissue (called the stasis zone) may spread out around the wound. In the stasis zone, tissue is hyperemic and inflammatory. When the skin's natural barrier is breached, microorganisms can enter the body and cause poor temperature regulation, fluid loss, and invasion. Intravascular volume loss is typically worsened by injured or edematous tissues. Significant heat loss may occur from the wounded dermis' lack of thermoregulation, particularly in exposed wounds. The severity determines the different treatments. Serious burns require considerable care, while lesser burns just require cleaning and painkillers. Just-partially thickened burns must be cleansed with soap and water before being clothed. For full-thickness burns, surgery, including skin grafting, is frequently required. Extensive intravenous fluid doses are often required to treat serious burns resulting from tissue edema and capillary fluid leakage.

## Introduction and background

Burns are dreadful wounds that may happen to anybody, anywhere, at any time. The bulk of burn injuries are due to radiation from steaming liquids, solids, or flames; however, other elements such as friction, cold, radiation, heat, chemicals from nature, or electric sources may also play a role. All burn injuries result in tissue loss due to energy transfer, even if different causes might lead to different physiological and pathophysiological consequences. For example, a flame or boiling grease may create a major burn very fast, while scalding injuries, or burns from hot liquids or steam, initially manifest as injuries because heat and energy sources are rapidly diluted. While high pH chemicals (pH approximately 1) cause colliquative necrosis, which transforms the tissue into a fluid, gelatinous mass, acidic chemicals cause coagulation necrosis, which retains the dead tissue's molecular structure [1]. The optimal course of care for a burn injury is determined by its specific etiology. Treating severe thermal burns the same way as frostbite, for example, would be inappropriate. Alternatively, to treat severe thermal burns, thrombolysis may be used in combination with moist rewarming [2].

Burn injury occurs when skin is exposed to heat [3]. There are various forms of injuries caused by burns, and the extent of the burn injury on the body surface area affects the wound morbidity and mortality of the patient [4]. The precise site of the burn, the surrounding temperature, and the length of time the victim was exposed to the heat source all have an impact on the severity of the injury [5]. Burn injuries are becoming less prevalent in high-income countries, but they are still widespread in other countries; 90% of all burn cases occur in less and moderate-income regions. There are a total of 11 million injuries due to burns of all sorts annually, 180,000 of which can be fatal, according to the World Health Organization (WHO). Burn injuries occur at various rates [6].

## Review

Methodology

**Figure 1:**
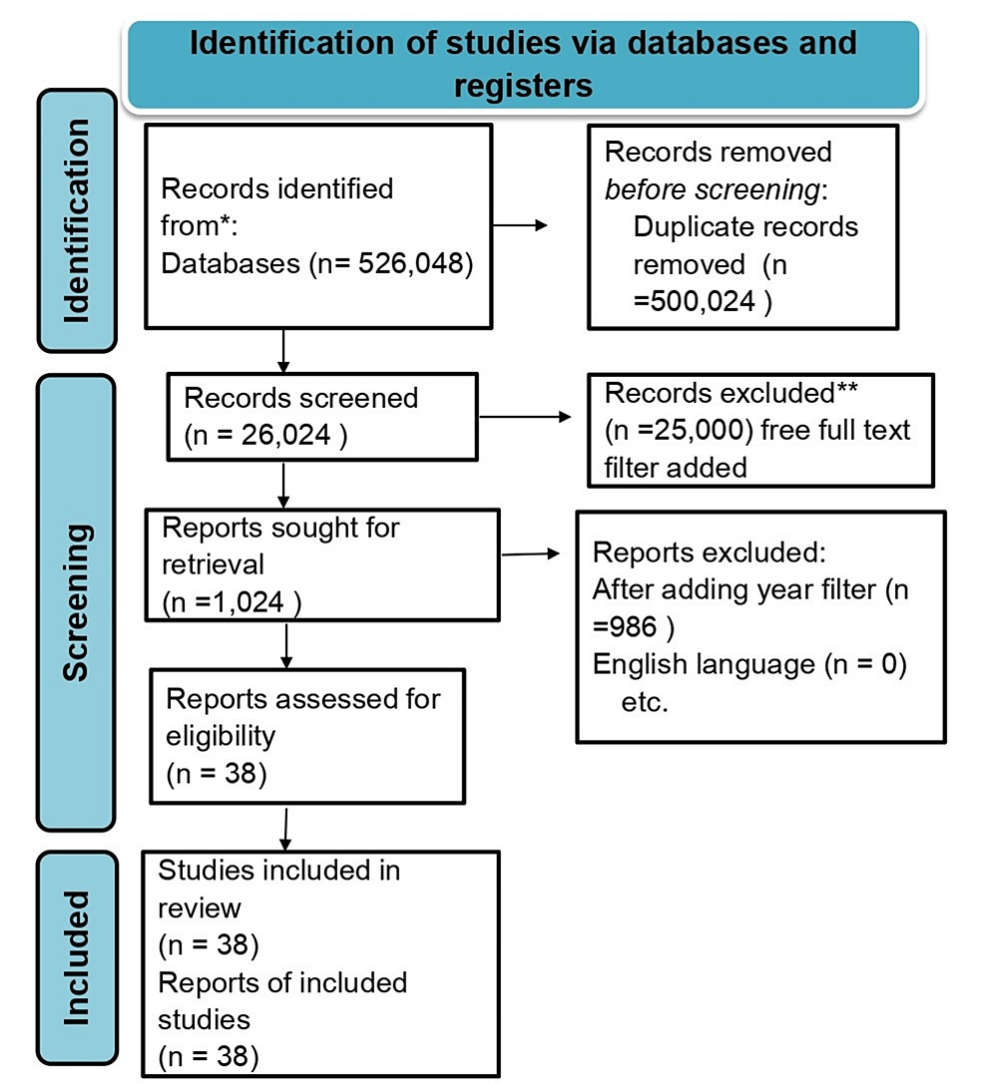
PRISMA Flow Diagram for Screening and Phasewise Management of Burn Injuries PRISMA: Preferred Reporting Items for Systematic Reviews and Meta-Analyses, n: number

We undertook a systematic search review through PubMed and CENTRAL in November 2020 using keywords such as "Screening and Phasewise Management of Burn Injuries" and "Management of Burn Injuries" ((Management of Burn Injuries[Title/Abstract]) OR ("Screening and Phasewise Management of Burn Injuries" [MeSH Terms]) AND ("Screening and Phasewise Management of Burn Injuries" [Title/Abstract]) OR (CEoSBP and Burn Injuries [Title/Abstract])). We also looked through the bibliographies of relevant literature to find significant sources. February 2022 saw an update to the search. Independently, one reviewer first checked the title and abstract of the retrieved papers against the inclusion criteria before moving on to the complete texts. About 20% of these papers were additionally examined by another reviewer to confirm that the studies were included (Figure 1).

Classification of burn injuries

When examining a burn, four elements must be taken into account: physical appearance, blanching under pain, pressure, and sensation. The American Burn Association criteria may be used to classify burns by thickness based on those four factors. Healing from burn wounds is typically a dynamic process. Some burns, especially partial-thickness burns, may get worse over the course of 2-4 days, peaking on day 3 [7].

According to their thickness, burns can be divided into "full-thickness" and "partial-thickness" categories. Recovery from a superficial partial-thickness burn, which only affects the epidermal and outer layer of the skin, takes just 10-14 days, and scarring is improbable. In addition to taking longer to cure (3-6 weeks), hypertrophic scarring is more likely to occur if the burn extends to deeper skin layers with more appendage damage. Full-thickness burns completely damage the skin's layers, necessitating surgical intervention to ensure proper wound healing [8].

The Rule of Nines

A quick way to evaluate the degree of a burn is to use the "rule of nines." Using this methodology, the body's surface area is divided into percentages. The head, neck, back, and front make up 9% of the body's total surface area. The front and back surfaces of each arm and hand make up approximately 9% of the body's surface area. The stomach (9%) and the chest (9%) make up the body's total surface area. The upper and lower backs each make up about 9% and 9% of the body's surface area, respectively. The back and front surfaces of each leg and foot combined make up 18% of the body's surface area. The vaginal area makes up about 1% of its surface.

Pathophysiology of burn injury

Research relating to clinical experiments have shown that within hours after injury, severe burns (regardless of the origin) trigger a severely imbalanced inflammation response in the host. Raised levels of chemokines, cytokines, and proteins related to the initial phase of treatment, along with a hypermetabolic state following a prolonged sympathetic response, which might remain past the acute duration during therapy, are characteristics of inflammation and stressful reactions [9].

**Figure 2:**
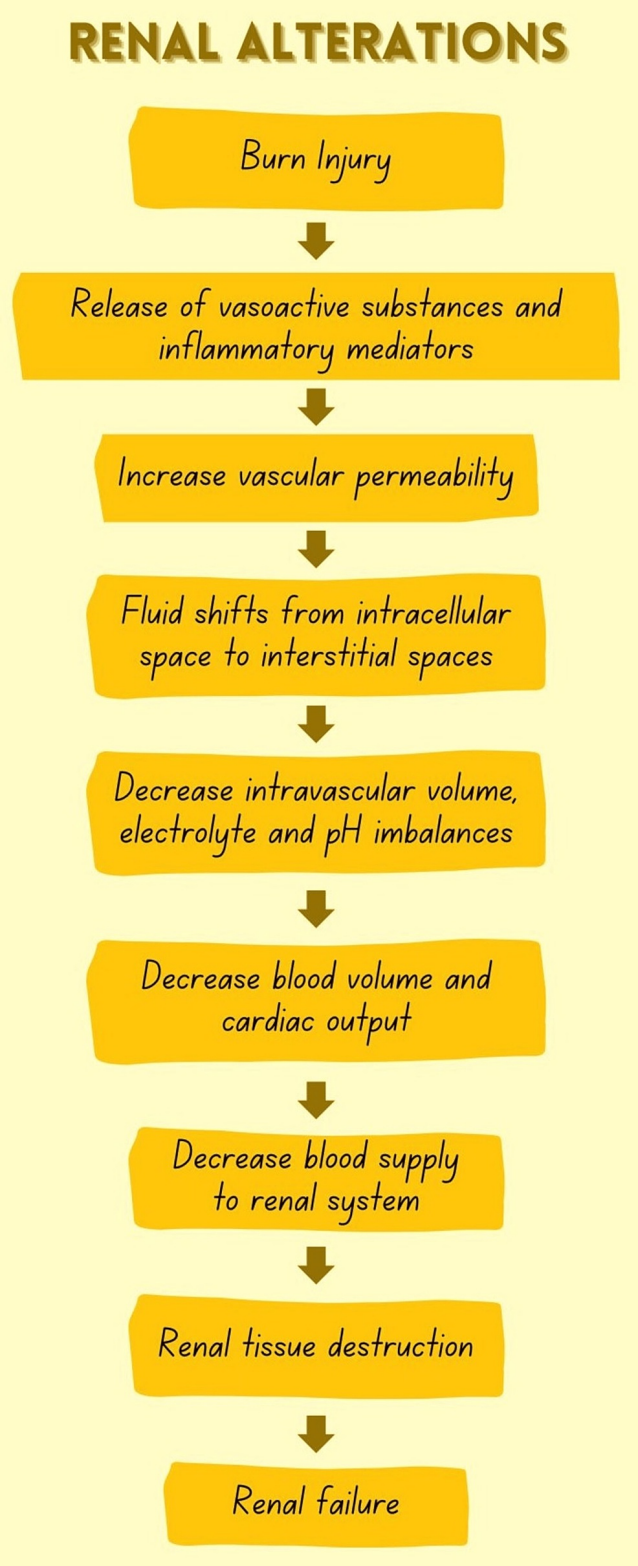
Renal Alterations in Burn Injuries Note: Author's own creation

The degree to which the host reacts is influenced by the intensity of the burn (a measurement of the total body surface area (TBSA) based on the severity of the burn), trigger, concurrent inhalation trauma, exposure to hazardous materials, other traumas, and patient-specific variables such as their ages, previous unresolved problems with health, use of alcohol or drugs, and the time spent looking for medical attention. The first reaction of the host to severe burns is similar to several other inflammatory conditions brought on by loss of tissue, such as major surgery or trauma, on the basis of the injury (Figure 2) [10].

Initial Injury

Following an injury, a burn wound is categorized into three zones: the most damaged zone, known as the zone of coagulation; the least damaged zone, known as the zone of ischemia or stasis; and the closest to the skin, known as the zone of hyperemia, which shows elevated inflammatory vasodilation. Depending on the area of the burn injury, there may be varying degrees of cellular damage. These might include cellular autophagy occurring immediately after the burn injury during the first 24 hours, delayed onset mortality occurring between 24 and 48 hours later, and varying levels of oxidative stress. The inflammatory phase, which is dynamic and overlaps with later phases, is when these wounds naturally heal. Initially, monocytes and neutrophils are evacuated first [11]. The adrenal glands also release catecholamines and cortisone, two stress hormones with widespread effects, near the site of injury, along with other inflammatory mediators such as cytokines [12]. Distinct capillary leaks of fluid move intravascularly to the interstitial cavity, considerably increasing tissue swelling and fluid buildup, severely impeding oxygen supply and tissue perfusion. One typical physiological condition that arises from burn damage is distributed shock [13].

Following the initial 72-96 hour hypometabolic state, people with severe burns typically experience a state of hypermetabolism (also known as the flow phase). This is characterized by a decreased metabolic rate and volume of intravascular fluid, lowered tissue perfusion, and decreased cardiac output. It may be brought on by intracellular mechanisms such as elevated stress in the endoplasmic reticulum (ER) and mitochondrial dysfunction. The burn injury-related hypermetabolic state may persist for up to 36 months beyond the original damage [14].

In addition to causing hypovolemic and hypermetabolic reactions, burn injuries significantly impair immunity. Soon after a burn injury, immune cells such as macrophages, monocytes, and neutrophils get activated and begin searching for endogenous materials such as alarmins or damage-related molecular patterns (DAMPs), which are produced due to fire-mediated damage to tissue. Pathogen-associated molecular pattern molecules (PAMPs), which are exogenous equivalents of DAMPs, are recognized by pattern recognition receptors such as Toll-like receptors (TLRs) and NOD-like receptors (NLRs) [15].

Burn damage can also cause other organs to change or sustain injuries. One typical side effect of burns is inhalation injury, which is defined as damage to the respiratory tract or lung tissue and can be sustained by breathing in hot smoke or chemical by-products of ignition. Burns usually appear after inhalation damage and can vary in severity. Inhalation damage increases respiratory complications, the requirement for fluids, and death [16].

**Figure 3:**
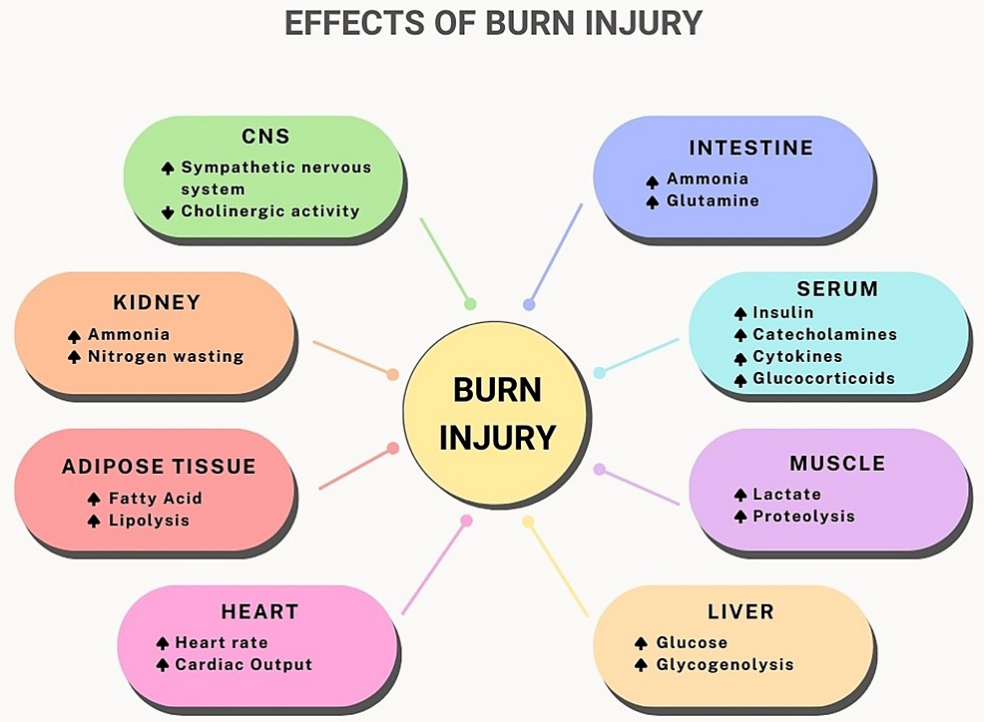
Effects of Burn Injuries on Various Organs CNS: central nervous system Note: Author's own creation

Concomitant trauma accounts for a relatively small percentage of burn injuries and often involves head trauma, fractures, peritoneal or pleural cavity injuries, or large soft tissue injuries (such as crush injuries or involving many tissue layers). The results for burn victims and other significant injury victims are often worse than for individuals who were not harmed (Figure 3) [17].

Diagnosis

The initial examination performed at the scene of the accident or in the emergency room of the hospital entails a quick, standardized assessment of the person's airway, ventilation, breathing, circulation, and cardiovascular health as well as an evaluation of their level of being exposed, impairment, neural deficit, and gross deformity (full undressing is required to facilitate the detection of any associated injuries). In order to avoid hypothermia during this examination, especially in small children and the elderly, a warm environment is required [18].

A preliminary examination of the burn's size is required since the quantity of oral or intravenous (IV) fluids needed for resuscitation relies on the burn's size (% of TBSA) [19,20].

A secondary evaluation is carried out when there are signs of further injuries or comorbidities, such as in the emergency department or burn center, and it typically involves laboratory tests and imaging studies. The subsequent survey also involves providing appropriate tetanus prophylaxis since burns are exposed wounds [21]. In individuals who have burn injuries that are not more than 15% TBSA, first laboratory tests include complete blood counts, electrolyte evaluations, coagulation profiles, and measurements of arterial blood gases. Normal levels of oxygen and radiographic images of the chest may not necessarily exclude the diagnosis in those who have a suspected smoke inhalation trauma as a lung inflammatory reaction can take some time for symptoms to appear [22].

Screening

The examination of a patient for burn injuries can be complicated since there are many physical, psychological, and emotional components to the injury, and our knowledge of functional restoration following damage is developing. In the case of an infection, for example, initial post-admit screenings for multidrug-resistant microorganisms can direct the choice of medications. Standardized prediction models can be used to estimate the likelihood that issues such as acute renal damage will arise. Which individuals require immediate intubation to prevent airway loss after smoke inhalation is yet unknown [23].

While burn injuries are more prevalent in males than in females, the prevalence of males is more often found in older age groups than in younger ones. In both categories, the most usually afflicted locations are the extremities. Hot drinks were the most frequent etiological agent in children, but electrical burns and workplace accidents were more common in the older group. Patients under the age of three constituted 62.4% of all cases among children. The percentage of total burnt body surface area, the need for an operation or critical care unit, the length of hospital stay, the need for blood transfusions or fresh frozen plasma, and the number of patients who passed away were all considerably greater in the adult group than in the pediatric group. However, the older group's neutrophil percentage was much greater, which might indicate an elevated inflammatory response.

Management

First aid procedures are advised for the timely treatment of more serious burn injuries as well as for all smaller burn injuries. The aforementioned main and secondary surveys are a part of the first phase of the acute care therapy of patients with more severe burns. Following a patient's admittance to a burn center, there are four main stages of care: resuscitation, burn injury coverings, critical clinical or supportive treatment, and rehabilitation. Outcome predictors are a crucial component in the planning of burn therapy.

The consistent response-dose interdependence between the burn's degree and its outcome is one of the traits that sets burn injury apart (i.e., the worse the result, the greater the burn size). The patient's age and the seriousness of the burn damage are both taken into consideration by the Baux score, which was originally introduced 50 years ago. The age of the patient and the size of the burn (% total body surface area (TBSA)) contribute equal roles in the Baux score, which can be used to predict mortality following a burn injury. The modified Baux score, the most common outcome predictor to date, may be used for patients of any age, including children, and it includes inhalation damage (present or absent) [24].

Phase I

A child patient suffering from burn injuries frequently follows the same treatment pathway as an adult patient. The concepts of both primary and secondary studies are the foundation of the initial strategy. However, since children's overall physiological reserves are lower, plus the head makes up a larger proportion of the TBSA, these two factors have a substantial role in the bulk of changes in how pediatric burn patients are treated. For instance, baby legs are smaller compared to newborns' larger head-to-body ratios. Children should also receive weight-based management fluids by IV 5% dextrose solution in normal saline solution because of the limited glycogen stores they carry. Comparing adult patients, treating hypermetabolism, dressing the wound, and developing long-term goals are the most important aspects of juvenile patient care. Individuals older than 65 years, who have the poorest outcomes after burn damage, are far more likely to have pre-existing fragility [25].

Elderly people display a specific response following burn injury, which causes reduced oxygenation and perfusion of organs. Additionally, malnutrition, psychological problems, and infections are all associated with higher rates of morbidity and death in the elderly population [17].

Phase II

Burn shock causes diffuse leaks of the capillary, which leads to increased intravascular volume reduction, mineral and plasma losses, obstruction of end-site perfusion, and cellular dysoxia. Burn shock integrates hypovolemic, distributive, and cardiac characteristics. Burn shock begins to appear during the first 48 hours following a burn injury due to a dysregulated inflammatory response. As was said earlier, a number of factors influence the amount of reaction, and the interaction of these elements creates considerable complexity that calls for a customized approach. Those who have burns larger than 20% TBSA usually require fluid resuscitation. However, if they additionally had electrical trauma, smoke inhalation, or contemporaneous trauma, those with less severe burns could still require fluid resuscitation. Maintaining end-organ perfusion is the primary goal of fluid resuscitation, while preventing adverse reactions such as extremity, abdominal, and orbital compartment syndromes is a supplementary objective [26].

A balanced crystalloid, most frequently warmed Ringer's lactate solution, is the first fluid of choice. Over-resuscitation condition, which is defined by continuous high-volume resuscitation of the crystalloid and links to the emergence of resuscitation-related morbidity, may affect a significant portion of patients suffering from major burns. The introduction of adjuncts in colloid rescue, such as albumin or plasma, has solved this problem [27]. By maintaining the glycocalyx cell wall, plasma seems to have specific advantages over various resuscitative fluids in maintaining the capillary endothelium. When compared to other blood products, plasma has different dangers, including lung damage transfusion. However, recent advancements in research have lessened the threat [28].

Due to vitamin C's antioxidants and reactive oxygen species-scavenging properties, its application in burn resuscitation has attracted attention. Initial animal tests showed favorable results, with less edema and capillary leakage, less lipid peroxidation, and less fluid demand as compared to crystalloid alone. Elevated dose of vitamin C lowered one-day crystalloid levels of fluid and improved several respiratory and oxygenation indices in a small uncontrolled study in humans, but there was no discernible change in mortality [29].

The removal of circulating inflammatory mediators is the aim of blood purifying techniques. Therapeutic plasma exchange (TPE) has revealed lowered lactate levels, increased mean arterial blood pressure, greater urine output, and lower resuscitative volume needs. Unfortunately, only a small number of facilities employ TPE since it is costly and requires a lot of resources and huge amounts of donor plasma or colloid for a single treatment [30].

Phase III

Until the middle of the 20th century, burn injuries were treated with high expectations. Tragically, however, many patients passed away from severe sepsis while they were waiting for the open wounds to heal secondary to the burn or for the eschar (scab) formed as a result of the burn to peel off. Because they delay septic development, topical antimicrobials have proven helpful in keeping patients with bigger, complex burns from developing sepsis. However, survival rates did not improve and hospital stays did not decrease until when grafting and early excision were implemented [31].

Prophylactic systemic antibiotics are not administered to patients who have had an acute burn. However, the mainstay of nonsurgical burn therapy has traditionally been topical antibiotics. Topical agents come in a variety of forms, such as liquids, lotions, ointments, and impregnated dressings. Since germs have a limited level of clinical resistance, dressing therapies often involve some kind of silver. Every burn unit's regional or localized location often selects a certain dressing based on staff preferences, previous experience, and availability. Nonetheless, there is no unambiguous agreement that one therapy is preferable to another since the literature endorsing one style of dressing over another is of varying caliber. Any dressing that is applied has to possess some kind of antibacterial properties [32].

Scarring is a major factor as a long-term result of burn injuries; scars should be flat and rarely discolored. Following burn injury, the scars are typified by increased action of collagenase enzyme, decreased expression of transforming growth factor (TGF), and mostly M1-phenotyped macrophages that support T helper 1 cell subsets. On the other hand, burns may leave a pathological scar. Deep burns that are either partial or full thickness heal more slowly, which raises the risk of pathological scarring, particularly if the burns are followed by a protracted acute inflammatory phase. Excessive collagen deposition produces pathological scars, which are thick, stiff marks that can hurt, itch, and contract, restricting function [33].

After thermal/heat/burn injury, keloids and hypertrophic scars are the two main subtypes of pathological scars that develop. Burn victims are more likely to have hypertrophic scars, which 30%-90% of them do. Hypertrophic scarring, which is often contained within the boundaries of the injury and does not reoccur after getting excised, is more likely to develop when wound healing is delayed (by more than three weeks). In hypertrophic scars, decreased collagenase activity perturbs collagen synthesis and breakdown, resulting in crosslinked collagen fiber bundles that are alongside the epidermal surface. Particularly, type III collagen is overproduced, while type I collagen expression is lowered [34].

Conversely, keloidal scars usually develop later after the initial incidence and are more common in individuals with dark skin color. Elevated fibroproliferative lesions consisting of twisted bundles of type I and type III collagen are known as keloid scars. Their characteristics are similar to tumors, and they use anaerobic glycolysis to make the metabolites needed for the proliferation of cells. Additionally, keloid scars exhibit uncontrolled expanding, encroaching onto healthy tissues, and reoccurring despite therapy [35]. Although the pigmentation of keloid scars may differ from normal skin in certain ways, it often exceeds the patient's "baseline" skin pigmentation. Both hypertrophic and keloid scars, which affect quality of life differently, are treated with a combination of intralesional medication therapy, contracture release, scar excision, scar massage, and laser therapy.

Phase IV

The goal of post-engraftment management should be to offer supportive intensive care that fosters ideal conditions for wound healing, as wound closure is linked to decreased instances of mortality and morbidity. The main goal of all aspects of care should be to promote wound healing while preventing frequently occurring hospital-acquired nosocomial complications, also known as nosocomial complications (such as venous thromboembolic problems, stress ulcers, pneumonia triggered by ventilator, catheter-related bloodstream infections, and infections associated with urinary tract catheters). To improve the conditions for wound healing, nutritional support and pharmacological adjuncts that reduce the effects of hypermetabolism and promote recovery (such as propranolol and oxandrolone), along with immediate hemodynamic support (resuscitation of fluid and occasional vasopressor support), are all required [36]. Effective management of pain is essential at all treatment stages, necessitating the prescription of opioids and other pain-relieving adjuncts.

Phase V

Even though the focus should be on clinical and surgical remedies, planning for recovery and rebuilding after serious burn damage should start as soon as a patient is admitted. Early multidisciplinary burn team engagement during hospitalization aids in maintaining the focus on "functional survival" [37]. Physical and occupational therapists on these teams apply rehabilitation techniques to every facet of burn treatment. Furthermore, in those who are receiving mechanical ventilation, simple measures such as appropriate limb positioning, splinting, and gradual weight-bearing can lessen burn contracture, reduce edema, and enhance functional results.

**Figure 4:**
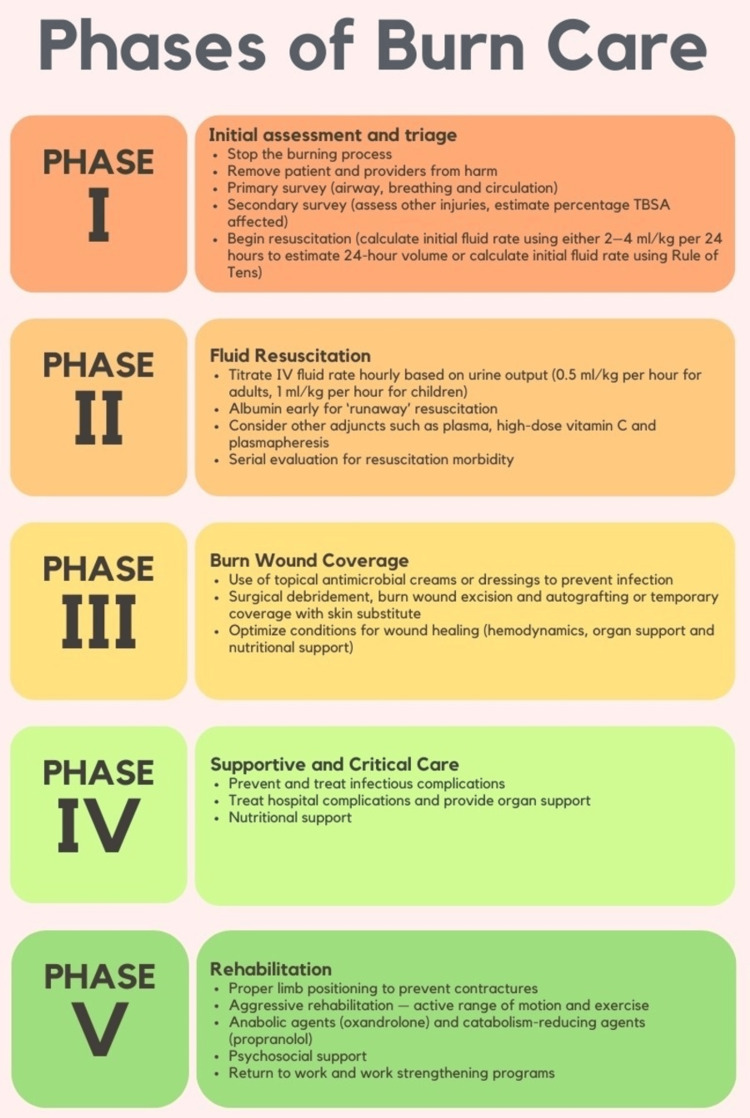
Phases of Burn Care TBSA: total body surface area, IV: intravenous

A multicenter observational research comprising 307 individuals suffering from acute burn injuries was the first to link therapeutic measures with positive patient outcomes. Longer treatment sessions were significantly associated with a decrease in burn wound contractures, according to the findings (Figure 4) [38].

## Conclusions

Everybody is prone to burn injuries, which are unpleasant, unplanned, and hazardous circumstances that might arise at any point in their life. They have an impact on a significant portion of people worldwide. Numerous factors vary, such as the degree of the injury, the patient's age, the cause of the injury, and the course of treatment. It is critical to get the treatment that one requires and check for injuries because of these unforeseen circumstances. Patients might undergo a long course of treatment since burn care involves many phases.
